# Balance and Weight Distribution over the Lower Limbs Following Calcaneal Fracture Treatment with the Ilizarov Method

**DOI:** 10.3390/jcm13061676

**Published:** 2024-03-14

**Authors:** Marcin Pelc, Krystian Kazubski, Wiktor Urbański, Paweł Leyko, Joanna Kochańska-Bieri, Łukasz Tomczyk, Grzegorz Konieczny, Piotr Morasiewicz

**Affiliations:** 1 Institute of Medical Sciences, University of Opole, Witosa 26, 45-401 Opole, Poland; 2 Department of Orthopaedic and Trauma Surgery, Institute of Medical Sciences, University of Opole, Witosa 26, 45-401 Opole, Poland; 3 Department of Neurosurgery, Wrocław Medical University, Borowska 213, 50-556 Wroclaw, Poland; 4 Universitätsspital CH, University of Basel, Petersgraben 4, 4031 Basel, Switzerland; 5 Department of Food Safety and Quality Management, Poznan University of Life Sciences, Wojska Polskiego 28, 60-637 Poznan, Poland; 6 Faculty of Health and Physical Culture Sciences, Witelon Collegium State University, Sejmowa 5A, 59-220 Legnica, Poland

**Keywords:** balance, weight distribution, biomechanics, Ilizarov method, intra-articular, calcaneal fractures

## Abstract

**Background**: The biomechanical outcomes of intra-articular calcaneal fracture treatment have not been fully explored. The purpose of this study was to analyze pedobarographic assessments of balance and body weight distribution over the lower limbs in patients following calcaneal fracture treatment with the Ilizarov method and to compare the results with those of a control group. **Materials and Methods**: The data for our retrospective study came from cases of intra-articular calcaneal fractures treated with the Polish modification of the Ilizarov method in the period between 2021 and 2022. The experimental group (21 patients; 7 women, 14 men) included Sanders classification calcaneal fractures type 2 (*n* = 3), type 3 (*n* = 5), and type 4 (*n* = 13). The control group comprised 21 sex-matched healthy volunteers, with no significant differences from the experimental group in terms of age or BMI. The examination included an assessment of balance and weight distribution over the lower limbs. The device used was a FreeMED MAXI pedobarographic platform (SensorMedica). **Results**: The mean displacement of the center of gravity in the experimental group was significantly higher at 1307.31 mm than in the control group (896.34 mm; *p* = 0.038). The mean area of the center of gravity was not significantly different between the groups. An analysis of weight distribution over the operated and uninjured limb in the experimental group and the non-dominant and dominant limb, respectively, in the control group revealed no significant differences. We observed no significant differences in the percentage of weight distribution over the lower limbs between the operated limb in the experimental group and the non-dominant limb in the control group, or between the uninjured limb in the experimental group and the dominant limb in the control group. **Conclusions**: The use of the Ilizarov method in calcaneal fracture treatment helps normalize the percentage weight distribution in the lower limbs, with the results comparable with those obtained in the healthy control group. The mean displacement of the center of gravity was worse in the experimental group than in controls; whereas the mean area of the center of gravity was comparable between the two groups. Treatment of calcaneal fractures with the Ilizarov method does not help achieve completely normal static parameters of lower-limb biomechanics. Patients treated for calcaneal fractures with the Ilizarov method require longer and more intense rehabilitation and follow-up.

## 1. Introduction

Fractures of the calcaneus account for approximately 2% of all fractures and for 50–60% of tarsal fractures [1,2,3,4,5]. The intra-articular and comminuted fractures of the calcaneus that require surgical treatment constitute approximately 75% of all calcaneal fractures [1,2,3,4]. There is no gold standard for the treatment of intra-articular and comminuted fractures of the calcaneus [1,2,3,4,6,7,8,9,10,11,12,13]. In the past, most calcaneal fractures were treated either by closed reduction and cast immobilization or by bone fragment repositioning and fixation with a few Kirschner wires or Steinmann pins [2,6,10]. Technological advancement has popularized the technique of open reduction and internal plate fixation of calcaneal fractures [1,2,3,4,6,7]; however, the necessary large incision has been associated with a high risk of complications, including delayed wound healing, infections, skin and soft tissue necrosis, fixation material-induced irritation, or loss of fixation (14–33%) [1,2,3,6,7].

One of the techniques used in calcaneal fracture management is the Ilizarov method [2,3,4,5,6,7,8,9,10,11,12,13,14]. Due to the high risk of complications and the complexity of the required surgical technique, calcaneal fractures have always posed a challenge for orthopedic surgeons [1,2,3,4,6,7,9,10,11,12,13]. Earlier papers on the topic dealt primarily with the clinical [2,3,4,5,6,7,10,12], radiological [2,3,4,5,9,10,13], and functional [2,3,6,9,10,13] outcomes of treating calcaneal fractures with external fixators and the Ilizarov method.

The growing use of various implants (Kirschner wires, Schanz pins) to complement the Ilizarov method may increase the risk of complications, such as peri-implant infections, delayed wound healing, or skin and soft-tissue necrosis [2,4]. The techniques for intra-articular calcaneal fracture management reported to date include the use of the Ilizarov method along with the insertion of at least three Kirschner wires into the foot [2,3,5,6,7,8,9,12,13,14]. The modified approach to intra-articular calcaneal fractures with the use of an Ilizarov fixator conducted in a center in Wrocław, Poland, requires the insertion of a single Kirschner wire into the foot [4].

The biomechanical outcomes of intra-articular calcaneal fracture treatment have not been fully explored. Such fractures result in bone fragment displacement, which alters the overall shape and three-dimensional structure of the calcaneus and of the whole foot [1,2,4,6]. One of the purposes of surgical treatment in intra-articular calcaneal fractures is to restore the shape and three-dimensional structure of both the calcaneus and the whole foot, in order to normalize kinetic and static parameters of the lower limbs [1,2,4,6]. Any abnormalities in the three-dimensional structure of the calcaneus and foot may lead to asymmetric load distribution in the foot, which causes pain, as well as accelerates tissue degeneration [4]. Post-traumatic deformities and changes in three-dimensional structure of the calcaneus and foot may adversely affect gait, balance, and weight distribution over the lower limbs [1,2,4,6,15,16,17,18,19,20,21,22,23,24,25].

Normal gait function is largely dependent on the anatomical bony structure of the foot [5,6,7,14]. Apart from the standard clinical and radiological assessments following lower-limb surgery, it is very important to also evaluate biomechanical parameters [15,16,17,18,19,20,21,22,23,24,25]. Pedobarography helps assess balance parameters and the distribution of loads on the lower limbs [15,16,17,18,19,20,21,22,23,26,27,28,29,30,31,32,33,34,35,36,37]. Pedobarography is an accepted method for examining the statics and dynamics of musculoskeletal issues [15,16,17,18,19,20,21,22,23,24,25,26,27,28,29,30,31,32,33,34,36]. Pedobarography is a useful, reproducible, objective, and comparable assessment method in the treatment of musculoskeletal pathologies [15,16,17,18,19,20,21,22,23,26,27,28,29,34,35,36]. Unfortunately, there is a lack of available literature on lower-limb biomechanics assessments following calcaneal fracture treatment with the Ilizarov method. The authors of earlier papers on calcaneal fracture treatment have only assessed gait following an open reduction and internal plate fixation approach [22,23,24,25]. The assessed parameters included also the mean contact area, peak pressures in the forefoot and hindfoot, and total contact time in patients with calcaneal fractures treated with an open reduction and internal plate fixation approach [33,34]. There have been no studies to assess the balance and weight distribution over the lower limbs following calcaneal fracture treatment. The studies conducted so far included assessments of balance and weight distribution over the lower limbs following lengthening and corrective corticotomy procedures on the thigh and leg with the Ilizarov method, ankle joint arthrodesis procedures, or tibial nonunion treatment with the Ilizarov method [15,16,17,18].

We hypothesized that calcaneal fracture treatment with the Ilizarov method would help restore normal balance and weight distribution over the lower limbs. The purpose of this study was to analyze pedobarographic assessments of balance and body weight distribution over the lower limbs in patients following calcaneal fracture treatment with the Ilizarov method and to compare the results with those of a control group of healthy individuals.

## 2. Materials and Methods

The data for our retrospective study came from patients with intra-articular calcaneal fractures treated with the Polish modification of the Ilizarov method in the period between 2021 and 2022. The study inclusion criteria were as follows: intra-articular calcaneal fracture treated with the Polish modification of the Ilizarov method, a follow-up period of over 2 years after treatment completion, complete medical and radiological records, complete pedobarographic assessment records, patient’s written informed consent, and the absence of lower-limb comorbidities. The study exclusion criteria were as follows: calcaneal fracture treatment with a method different than the Ilizarov method, a follow-up period of less than 2 years, incomplete medical and/or radiographic records, incomplete pedobarographic assessment records, other lower-limb injuries, lower-limb comorbidities, and a lack of consent. All patients were informed of the voluntary nature of study participation and the possibility of withdrawing from the study at any time. This study was approved by the local ethics committee (UO/0023/KB/2023).

Application of the inclusion and exclusion criteria yielded 21 patients (7 women, 14 men), aged from 25 to 67 years (mean age 47 years), with a body mass index of 24–40 (mean 28), height of 152–188 cm (mean 171 cm), body weight of 61–130 kg (mean 81 kg). The control group comprised 21 sex-matched healthy volunteers, with no significant differences from the experimental group in terms of age, demographics, BMI, or physical activity levels.

The experimental group included Sanders classification calcaneal fractures type 2 (*n* = 3), type 3 (*n* = 5), and type 4 (*n* = 13). Each of the evaluated patients was operated on by the same surgeon, who used the Polish modification of the Ilizarov method for calcaneal fracture treatment [4] (verbal accounts by P. Koprowski and L. Morasiewicz).

**Figure 1:**
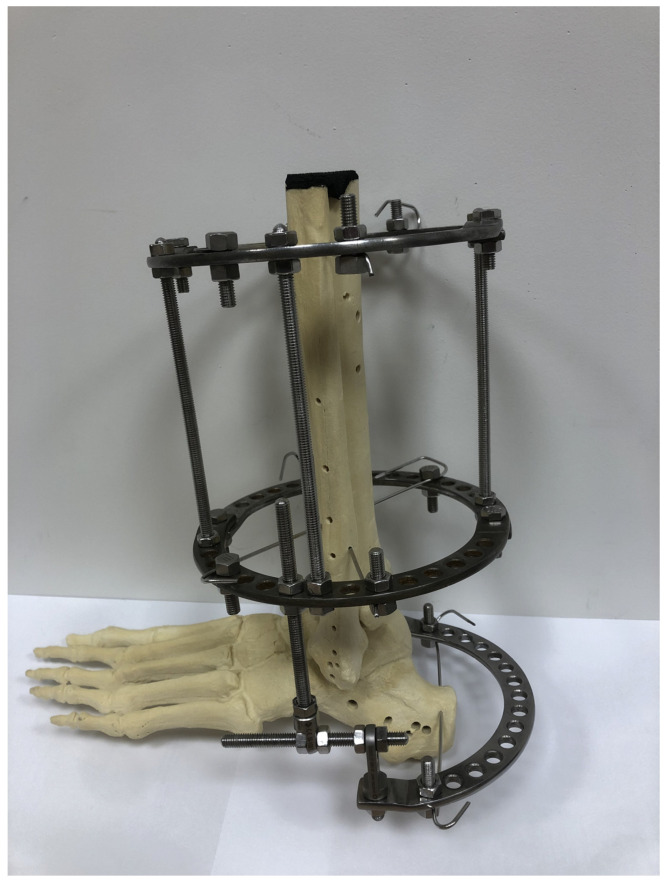
A three-dimensional model of the Polish modification of an Ilizarov fixator for calcaneal fracture treatment.

The external fixator used for calcaneal fracture treatment in accordance with the Polish modification of the Ilizarov method was composed of two fully circular rings, which were fixed to crural bones with Kirschner wires, and one half-ring, which was fixed to the calcaneus with a single Kirschner wire (Figure 1).

**Figure 2:**
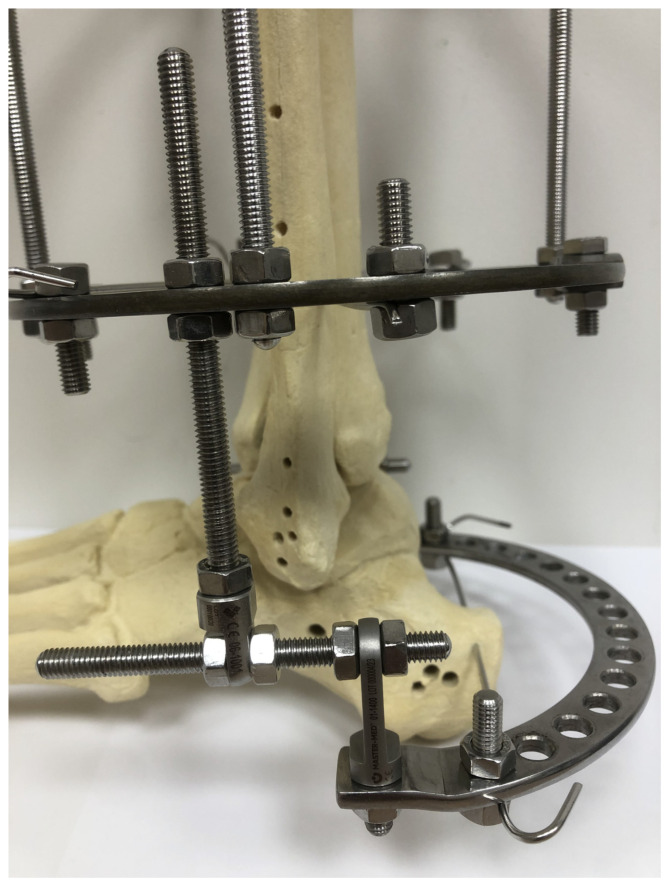
Detailed structure of an Ilizarov fixator.

All surgical procedures were conducted with a closed approach, without an open access to the calcaneus. Once the two full rings were mounted on the leg, one Kirschner wire was inserted (under fluoroscopy) into the calcaneal bone fragment that was both the most proximal and the most dorsal. Subsequently, the half-ring was positioned behind the foot and fixed to a Kirschner wire inserted into the calcaneus. The half-ring was then connected with the distal leg ring by means of two connectors (Figure 2).

Each connector was composed of two perpendicular, threaded rods (Figure 2). Once the fixator was mounted on the leg and foot, the calcaneal fracture was reduced under fluoroscopy. Ligamentotaxis via this modified Ilizarov fixator allowed a closed, indirect reduction in the calcaneal fracture. On day one after surgery, the patients began walking with two elbow crutches, with partial weight bearing on the treated limb. Gradually, the patients were allowed to bear more and more weight on the operated foot, to the extent of their pain tolerance. All patients underwent the same rehabilitation protocol and were scheduled for periodic follow-up visits in an outpatient setting. The follow-up visits included clinical examination and radiological imaging. The fixator was removed once clinical and radiological evidence of bone union was observed.

**Figure 3:**
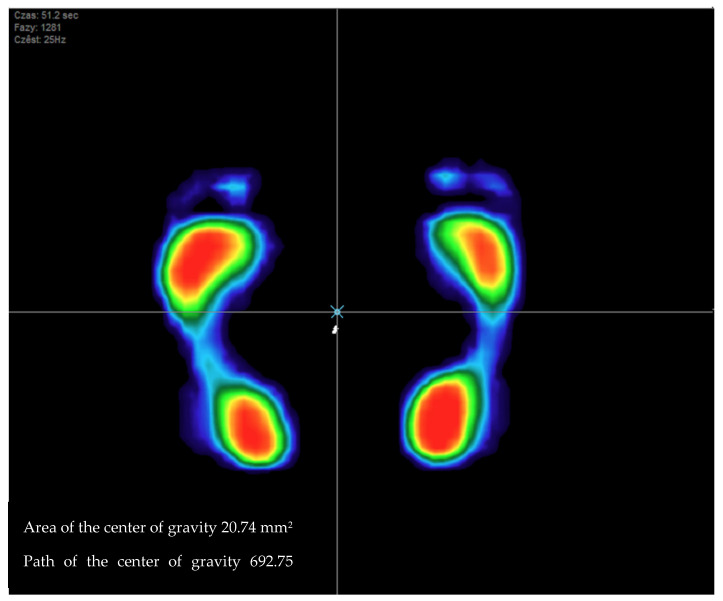
Image of balance test.

**Figure 4:**
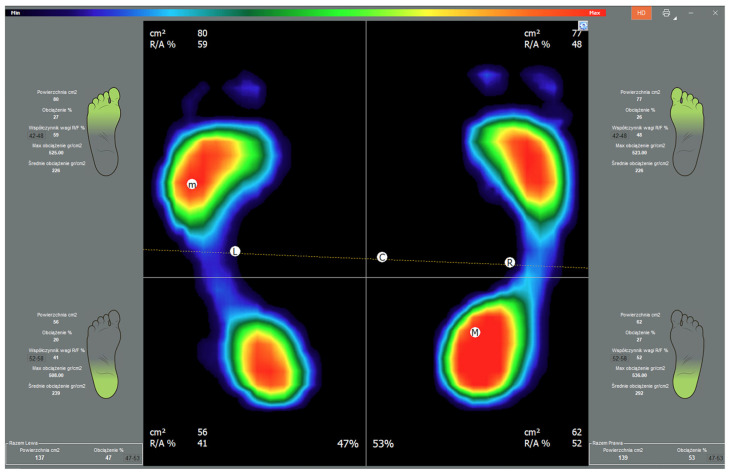
Image of the percentage weight distribution over the limbs. Color map of the pressures: red to dark green—from the area of the highest to the lowest level of pressure; blue—foot perimeter.

The clinical examination included assessments of balance (Figure 3) and weight distribution over the lower limbs (Figure 4).

**Figure 5:**
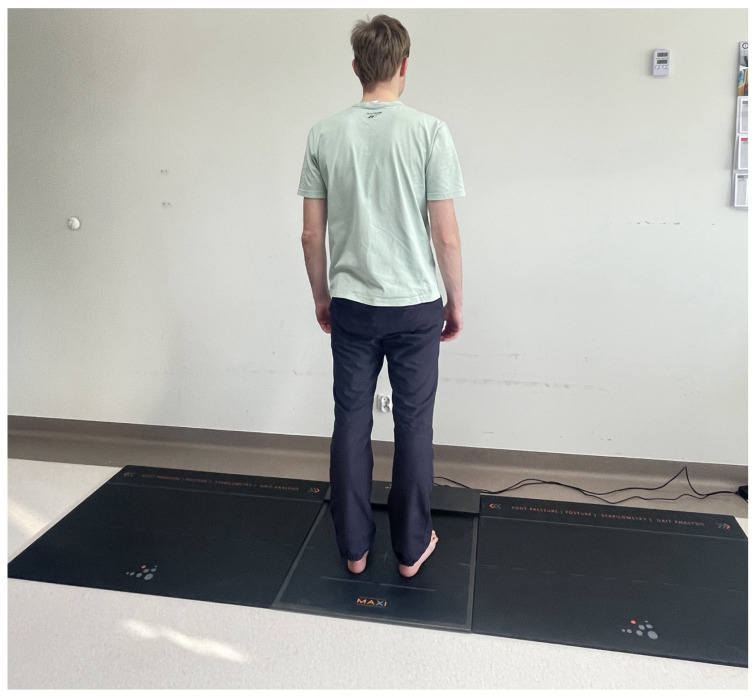
SensorMedica pedobarographic platform.

The device used was a FreeMED MAXI pedobarographic platform manufactured by SensorMedica (Guidonia Montecelio, Rome, Italy). The pedobarographic assessment set includes a platform measuring 63.5 × 70 cm (total active sensor area of 50 × 60 cm), two inactive mats measuring 70 × 100 cm each, and a computer with appropriate software, Figure 5.

The platform can measure pressures of up to 150 N/cm^2^ with a minimum acquisition frequency of 300 Hz in real time. The 3000 square resistive sensors coated in 24-carat gold, each with a durability of 1,000,000 cycles, ensure high accuracy and reproducibility of measurements [26,27,28,29,35,36].

Each study subject had received detailed instructions on the measurement procedure. During pedobarographic and posturographic assessments, each subject was asked to make corrective adjustments to his or her posture. The measurements were taken while the subjects had their eyes open and were standing on both lower limbs, with their feet positioned freely in a physiological position (with an external rotation of 5–10°) [30]. The mean duration of balance assessments was 51.2 s. Weight distribution was recorded following a 5-second stabilization after a subject stepped onto the platform. The subjects were advised to maintain an upright posture, with their arms hanging symmetrically along the torso, and to keep their eyes fixed on one point on the wall in front of them at their eye level. Each subject underwent the measurement three times, and the mean value of the three was used in further analyses. The measurements were recorded via FreeSTEP software, V.2.02.006. Subsequently, the results were exported onto a spreadsheet and analyzed statistically.

Balance was assessed based on center-of-gravity displacement. This parameter was expressed as the total distance (in millimeters) traversed by the center of gravity over the course of the evaluation [15,16,17,27]. Balance assessment was also based on the surface area determined by maximum displacements of the center of gravity and defined as the area (in mm^2^) enclosed by the points of maximum center-of-gravity displacement in all directions over the course of the evaluation [15,16,17,27].

Weight distribution over the lower limbs was expressed in percentage values. In the experimental group, we assessed the load on the uninjured and the treated lower limb and calculated the proportion of weight distribution on the forefoot and hindfoot of either limb. The dominant limbs in the control group of healthy individuals were compared with the uninjured limbs of treated individuals, and the non-dominant limbs of control individuals were compared with the operated limbs of treated individuals [15,16,18]. The results obtained in the experimental group of patients with calcaneal fractures treated with the Ilizarov method were compared with those obtained in the control group of healthy volunteers.

### Statistical Analysis

Data were statistically analyzed using Statistica 13.1. The Shapiro–Wilk test was used to check for normality of distribution. Continuous variables were reported as mean (±SD). A Levene’s test was performed to assess the homogeneity of variance within the two repeat sets of measurements. Inter-group comparisons of continuous variables were made with Student’s *t*-test. The level of statistical significance was set at *p* < 0.05.

## 3. Results

**Figure 6:**
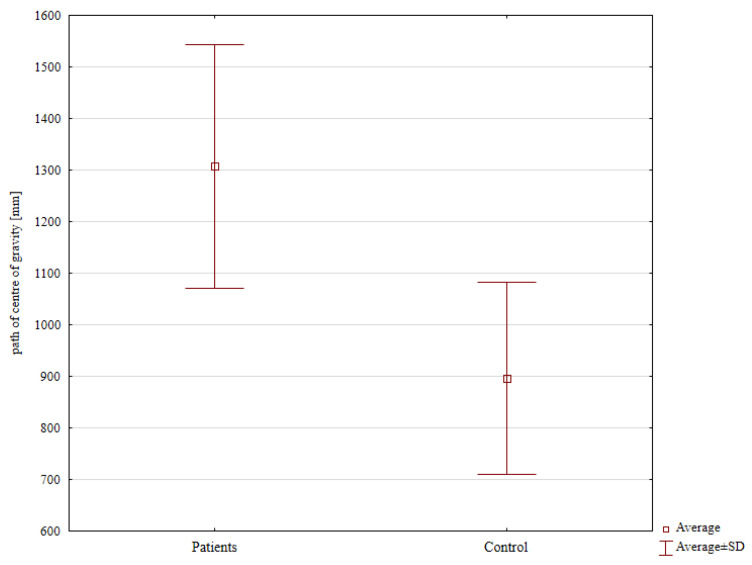
Path of the center of gravity in the experimental group compared with that in the control group.

The mean displacement of the center of gravity in the experimental group was significantly higher at 1307.31 mm than in the control group (896.34 mm; *p* = 0.038), (Figure 6, Table 1). The mean area of the center of gravity was 162.77 mm^2^ in the experimental group and 96.67 mm^2^ in the control group. This difference between groups was not statistically significant (Table 1).

An analysis of weight distribution over the operated and uninjured limb in the experimental group and the non-dominant and dominant limb, respectively, in the control group revealed no significant differences (Table 2).

**Figure 7:**
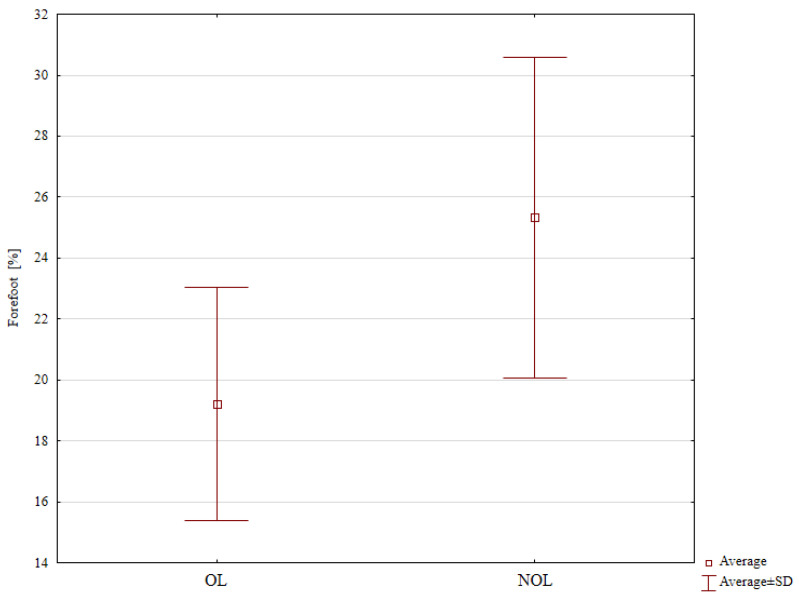
Weight distribution in the forefoot of the operated and the uninjured limbs.

Nonetheless, it is worth noting that patients treated with the Polish modification of the Ilizarov method tended to bear significantly less weight on the forefoot of the operated limb (19.22%) in comparison with that of the uninjured limb (25.33%), *p* = 0.038 (Table 2, Figure 7). We observed no significant differences in the proportion of weight borne on the hindfoot in the two study groups (Table 2).

**Figure 8:**
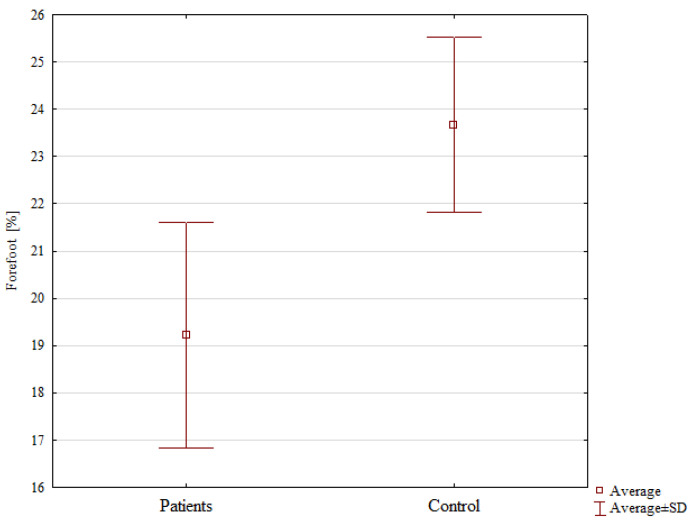
A comparison of weight distribution in the forefoot of the operated limb in the experimental group and that of the non-dominant limb in the control group.

The forefoot of the operated limbs in the experimental group also bore significantly less weight (19.22%) than that in the non-dominant limbs in the control group (23.66%), *p* = 0.026, (Table 3, Figure 8).

We observed no significant differences in the percentage of weight distribution between the operated limb in the experimental group and the non-dominant limb in the control group, or between the uninjured limb in the experimental group and the dominant limb in the control group (Table 3). Moreover, these compared pairs of limbs showed no significant differences in terms of any other analyzed parameters (Table 2 and Table 3).

## 4. Discussion

This paper presents our assessment of balance and weight distribution over the lower limbs following calcaneal fracture treatment with the Polish modification of the Ilizarov external fixator. We observed no differences in the percentage distribution of weight over the lower limbs between any of the following pairs of compared limbs: the operated and uninjured limbs in the experimental group; the operated limb in the experimental group and the non-dominant limb in the control group; and the uninjured limb in the experimental group and the dominant limb in the control group. The analysis of balance showed some of the results to be significantly poorer in the group of calcaneal fracture patients than in the group of healthy volunteers, which partly supports our hypothesis. The mean displacement of the center of gravity in the experimental group was not as good as that in the control group, whereas the mean area of the center of gravity was comparable in both groups.

Intra-articular calcaneal fractures often pose a challenge for orthopedic surgeons due to the complexity of the required surgery and high rates of complications [1,2,3,4,6,7,9,10,11,12,13,22,33,34]. The Ilizarov method has been adopted as one of the techniques used in the treatment of calcaneal fractures [2,3,4,5,6,7,8,9,10,11,12,13,14].

The goal of surgical treatment of intra-articular calcaneal fractures is to reduce pain and restore the three-dimensional structure of the calcaneus and the function of the foot [1,2,4,5,6,7,22].

Calcaneal fractures may lead to a lowered longitudinal arch, which results in flatfoot [23]. Some authors suggest that the normal shape and restored anatomical structure of the calcaneus determines normal lower-limb biomechanics and gait efficiency [1,2,4,6]. However, other authors reported good clinical and functional outcomes with poor radiological outcomes [1], and others reported poor clinical or functional outcomes with good radiological outcomes [2,7]. Achieving normal musculoskeletal biomechanics—including balance and weight distribution over the lower limbs—following treatment of musculoskeletal pathologies is possible in the case of normal ranges of motion, absence of pain, and restored bone anatomy [15,16,17,18,19,20,21,22,23,24]. Typically, weight distribution over the lower limbs is symmetrical [16,17]. In light of the above, it is important not only to conduct clinical and radiological assessments but also to assess balance and weight distribution over the lower limbs, as it is performed in analyzing treatment outcomes in various musculoskeletal pathologies, including injury-induced ones [15,16,17,18,19,20,21,22,23,24,25,31,32,33,34,36]. Abnormal biomechanical parameters, including balance and distribution of weight over the lower limbs, may indicate postoperative pain, limited range of motion, and decreased muscle strength, hence the great importance of lower-limb biomechanics assessments following treatment [15,16,17,18,19,20,21,22,23,24,25,31,32,33,34,36].

There have been no studies to assess lower-limb biomechanics following the treatment of calcaneal fractures with the Ilizarov method. Authors of earlier studies on gait reported abnormal gait parameters following calcaneal fractures treated with open reduction and internal plate fixation [22,23,24,25]. Some authors reported no differences between the treated and the uninjured limbs in terms of the mean contact area in the forefoot and hindfoot in patients after calcaneal fracture treatment with an open reduction and internal plate fixation approach but they assessed neither balance parameters nor percentage weight distribution over the lower limbs [34]. The group of patients who received conservative treatment for calcaneal fractures exhibited abnormal biomechanics between the treated and the uninjured limb in terms of the mean contact area in the forefoot and hindfoot [34]. Other authors reported differences between the treated and the uninjured limbs in terms of maximum pressure and total contact time in patients with calcaneal fractures treated with internal plate fixation [33]. There have been no reports of assessing balance and weight distribution over the lower limbs following calcaneal fracture treatment.

Theoretically, the Ilizarov method is more effective in restoring balance and weight distribution than other available treatments for calcaneal fractures (such as open reduction and fixation with a plate or screws). In comparison with other techniques of calcaneal fracture fixation, the Ilizarov method is less invasive, requires only a small incision, and is associated with a lower risk of infections and other complications [1,2,3,4,6,7,9,10,11,12,13]. In comparison with calcaneal fracture fixation with a plate or screws, the Ilizarov method allows patients to bear weight on the operated limb sooner and initiate intensive rehabilitation sooner than with other treatment methods.

Pajchert-Kozłowska et al. used a pedobarographic platform to assess balance in patients following treatment of tibial nonunion with the Ilizarov method [15]. Those authors reported the balance parameters in the experimental group to be comparable with those in healthy volunteers [15]. Another study, which evaluated patients following lower-limb corticotomy procedures with the Ilizarov method, showed poorer balance values in comparison with those in the healthy control group [16]. Analysis of balance following ankle joint arthrodesis with internal fixation or with external fixation with the Ilizarov method showed worse results in the group with internal fixation [17]. Rongies used a pedobarographic platform to assess 21 patients with coxarthrosis and reported balance improvement following rehabilitation [19].

In our group of patients, center-of-gravity displacement was significantly greater than that in the control group of healthy individuals. The area of the center of gravity in the experimental group was greater, though not significantly, than that in the control group. This suggests a lack of balance normalization following calcaneal fracture treatment with the Ilizarov method. Calcaneal fractures may result in swelling, reduced muscle strength, pain, and a limited range of motion [22,24], which may have adversely affected the balance in our experimental group. The balance parameters in our patients were comparable with those reported by authors who assessed patients after corticotomies using the Ilizarov method and after ankle joint arthrodeses using the Ilizarov method [16,17]. The fact that some balance parameters remained abnormal after calcaneal fracture treatment with the Ilizarov method indicates the need for a longer rehabilitation period and exercises for these patients.

In another group of 57 patients treated with lower-limb croticotomy with the Ilizarov method, there were no differences in the percentage weight distribution over the lower limbs between the operated and non-operated limb, and the absolute load values were comparable with those obtained in the healthy control group [16]. Analysis of percentage weight distribution over the lower limbs in patients treated with ankle joint arthrodesis with internal fixation and in those treated with an external Ilizarov fixator revealed no differences between the two groups in terms of weight distribution between the operated and the uninjured limb [17]. Pawik et al. assessed patients with tibial nonunion treated with the Ilizarov method [18]. Those authors observed no differences in the percentage weight distribution between the forefoot and hindfoot of either the operated and uninjured limb in the experimental group or between the experimental and control groups [18]. Güven et al. analyzed 37 patients who underwent surgical treatment of transtrochanteric femoral fractures with partial hemiarthroplasty or proximal femoral nail [31]. Using a pedobarographic platform, those authors assessed the differences in weight distribution between the operated and uninjured limbs in static conditions. The results showed a greater load on the uninjured limb in both analyzed groups [31]. Out of the 26 patients with isolated tarsometatarsal (Lisfranc) joint injuries evaluated by Shepers et al., one-half received surgical treatment and the other half received conservative treatment [32]. Study results showed both groups to have similar percentage weight distribution over the lower limbs. In the case of the injured foot, there was a significantly greater weight distribution on the posterior part of the foot than on the forefoot [32]. Tarczyńska et al. conducted a balance study on 30 patients, assessing the long-term effects of surgical treatment of Achilles tendon injury [36]. They compared two groups of patients: one who sought treatment within 4 weeks of the injury and the other who sought treatment after 4 weeks. Their results showed that delayed treatment of Achilles tendon injury leads to deterioration of balance parameters in long-term follow-up [36].

A fracture reduction that recreates the anatomical structure of the calcaneus helps restore the normal biomechanic parameters and three-dimensional structure of the foot and gain efficiency [1,2,4,6]. Our study showed a symmetrical percentage weight distribution between the operated and the uninjured limb in the experimental group. Similarly, we observed no differences in weight distribution between the operated limb in the experimental group and the non-dominant limb in the control group or between the uninjured limb in the experimental group and the dominant limb in the control group. The only statistically significant difference was in terms of forefoot loading, which was significantly lesser in the operated than in the uninjured limb in the experimental group. This indicates a normalization of percentage weight distribution over the lower limbs following fracture treatment with the Ilizarov method. The patients who underwent calcaneal fracture treatment with the Polish modification of the Ilizarov method achieved comparable percentage values of weight distribution over the lower limbs to those in the control group of healthy volunteers. The results of weight distribution over the lower limbs observed in our study are comparable with those reported in the literature [16,17,18].

One limitation of our study is its retrospective nature. This is due to the nature of injuries since patients with calcaneal fractures cannot undergo a normal pedobarographic assessment prior to treatment. Other authors also presented retrospective analyses of patients following calcaneal fracture treatment and retrospective pedobarographic analyses [3,4,5,6,8,9,10,12,13,14,15,16,17,18,22,23,24,25,31,33,34]. Another limitation of our study is the relatively small sample size. This is due to the low incidence of calcaneal fractures and the time constraints for pedobarographic assessments. However, many other authors assessed comparable or even smaller study groups [3,4,5,6,8,9,10,12,13,14,15,18,19,20,23,25,32,33,34,36]. One of the strengths of our study is the sex-, age-, and BMI-matched control group, a uniform rehabilitation protocol, the follow-up period of over 2 years, and all procedures being conducted by the same surgeon. In the future, we are planning to conduct similar studies in a larger patient population with a longer follow-up period and to assess gait parameters in patients with intra-articular calcaneal fractures treated with the Ilizarov method. We believe it is important to compare the balance parameters and percentage weight distribution over the lower limbs in patients following calcaneal fracture treatment with different fixation techniques (i.e., an external Ilizarov fixator vs. open reduction and internal fixation with a plate and screws). Our study showed that normal balance parameters were not restored following treatment; however, they were similar to those achieved by other patients following treatment with an Ilizarov fixator [16,17]. The fact that some balance parameters did not reach their normal values in our patients may be due to pain, a limited range of motion, swelling, and reduced muscle strength [15,16,17,18,19,20,21,22,23,24,25,31,32]. We are planning to conduct studies to assess the severity of pain, joint range of motion, muscle strength, and quality of life in patients following calcaneal fracture treatment with the Ilizarov method.

Our study showed that some balance parameters did not reach their normal values following calcaneal fracture treatment with the Ilizarov method. We believe that more attention should be paid to patient rehabilitation following calcaneal fracture treatment with the Ilizarov method. These patients should undergo a longer and more intense rehabilitation and have a longer period of follow-up visits. A longer period of post-treatment analgesia and exercises should be considered for patients following calcaneal fracture treatment with the Ilizarov method. Implementing these measures may help reduce pain and swelling and improve range of motion and muscle strength, which would restore normal biomechanical parameters in patients following calcaneal fracture treatment with the Ilizarov method.

## 5. Conclusions

The use of the Ilizarov method in calcaneal fracture treatment helps achieve normalization of percentage weight distribution in the lower limbs, with the results comparable with those obtained in the healthy control group.

Following treatment, calcaneal fracture patients showed worse mean displacement of the center of gravity than that in the control group, with no differences between these two groups in the mean area of the center of gravity.

Treatment of calcaneal fractures with the Ilizarov method does not help achieve completely normal static parameters of lower-limb biomechanics.

Patients with calcaneal fractures treated with the Ilizarov method require longer and more intense rehabilitation and follow-up periods.

## Figures and Tables

**Table 1:** Path of center of gravity and area of the center of gravity.

Analyzed Variable	Patients	Control Group	*p*-Value *
	Mean ± Standard Deviation		
Area of the center of gravity [mm^2^]	162.77 ± 132.85	96.67 ± 73.89	0.324
Path of the center of gravity [mm]	1307.31 ± 372.33	896.34 ± 272.89	0.038

* Student’s *t*-test.

**Table 2:** Body weight distribution in patients after treatment and in controls.

Loads on Limb	Control Group	Patients after Surgery
	Mean ± Standard Deviation	
OL [%]	47.16 ± 2.97	46.01 ± 5.67
NOL [%]	52.83 ± 13.72	53.11 ± 7.23
*p*-value *	0.715	0.077
OL forefoot [%]	23.66 ± 3.7	19.22 ± 4.79
NOL forefoot [%]	26.41 ± 4.75	25.33 ± 6.57
*p*-value *	0.128	0.038
OL hindfoot [%]	23.5 ± 3.06	27.66 ± 6.34
NOL hindfoot [%]	26.41 ± 4.81	27.77 ± 4.54
*p*-value *	0.090	0.966

OL—operated limb; NOL—non-operated limb. * Student’s *t*-test.

**Table 3:** Body weight distribution in the two groups.

Analyzed Variable	Patients	Control Group	*p*-Value *
	Mean ± Standard Deviation		
OL [%]	46.01 ± 5.67	47.16 ± 2.97	0.668
NOL [%]	53.11 ± 7.23	52.83 ± 13.72	0.390
OL forefoot [%]	19.22 ± 2.79	23.66 ± 2.71	0.026
OL hindfoot [%]	27.66 ± 5.34	23.5 ± 3.06	0.060
NOL forefoot [%]	25.33 ± 6.57	26.41 ± 4.75	0.666
NOL hindfoot [%]	27.77 ± 4.54	26.42 ± 4.81	0.519

OL—operated limb; NOL—non-operated limb. * Student’s *t*-test.

## Data Availability

The data presented in this study are available on request from the corresponding author.
